# Directed evolution strategies for improved enzymatic performance

**DOI:** 10.1186/1475-2859-4-29

**Published:** 2005-10-07

**Authors:** Edward G Hibbert, Paul A Dalby

**Affiliations:** 1 The Advanced Centre for Biochemical Engineering, Department of Biochemical Engineering, University College London, Torrington Place, London WC1E 7JE, UK

## Abstract

The engineering of enzymes with altered activity, specificity and stability, using directed evolution techniques that mimic evolution on a laboratory timescale, is now well established. However, the general acceptance of these methods as a route to new biocatalysts for organic synthesis requires further improvement of the methods for both ease-of-use and also for obtaining more significant changes in enzyme properties than is currently possible. Recent advances in library design, and methods of random mutagenesis, combined with new screening and selection tools, continue to push forward the potential of directed evolution. For example, protein engineers are now beginning to apply the vast body of knowledge and understanding of protein structure and function, to the design of focussed directed evolution libraries, with striking results compared to the previously favoured random mutagenesis and recombination of entire genes. Significant progress in computational design techniques which mimic the experimental process of library screening is also now enabling searches of much greater regions of sequence-space for those catalytic reactions that are broadly understood and, therefore, possible to model.

Biocatalysis for organic synthesis frequently makes use of whole-cells, in addition to isolated enzymes, either for a single reaction or for transformations via entire metabolic pathways. As many new whole-cell biocatalysts are being developed by metabolic engineering, the potential of directed evolution to improve these initial designs is also beginning to be realised.

## Introduction

Natural enzymes can catalyse reactions with up to 10^17 ^fold rate accelerations [1], and with exquisite control of regio- and stereo-chemistry. This along with their compatibility with mild aqueous conditions has led to their increasing use as biocatalysts in synthetic chemistry, especially in cases where chemical routes are difficult to implement [2,3]. Enzymes used in such biotransformations are frequently prepared as isolated enzymes in solution or immobilised onto resins, and used in the presence of organic solvents, harsh chemical compounds, or under conditions of temperature or pH that are suboptimal for enzyme activity. Such non-natural conditions also often result in poor enzyme activity, or complete deactivation due to denaturation or chemical modifications. Developments in protein engineering over the past ten years have enabled enzymes to be evolved *in vitro *for properties that favour the required process conditions, and also to obtain enzyme variants with altered substrate specificity or enantioselectivity [4,5]. Despite the significant advances to date on many industrially relevant enzymes, there still remains a need to improve directed evolution strategies and develop generic screening or selection tools which make the process of identifying novel enzyme activities more efficient, and also to access much greater changes to enzyme function.

Just as Nature evolves enzymes without any 'knowledge' of enzyme structure and function, the techniques of *in vitro *directed evolution mimic natural processes such as random mutagenesis [6] and sexual recombination [7-10] to improve enzymes without understanding them in great detail.

This review discusses recent advances in the techniques and strategies used for the directed evolution of biocatalytic enzymes, including the development of new genetic and computational strategies aimed at improving the quality and potential of enzyme libraries, as well as new screening tools that broaden the range of targets for directed evolution. Also discussed is the use of directed evolution for enhancing metabolically engineered pathways, and some future applications arising from novel pathway engineering in *E. coli*. Examples of the application of established methods to new enzymes are not discussed.

## New mutagenic strategies for directed enzyme evolution

Despite the rapid growth of published examples of directed evolution, there is still a clear need for alternative and improved methods for the directed evolution of enzymes. Current constraints include the difficulty in optimising ligation steps when large libraries (>10^6 ^variants) are sought for selection-based methods, practical limitations to library sizes that can be screened, and barriers to technology licensing [5]. Some improvements have been made in circumventing the need for ligations by adopting PCR-based approaches [11], and more recently by directing *in vivo *hypermutation with B cells to target genes delivered by retroviral infection [12]. However, ligation free approaches for DNA shuffling have yet to be demonstrated. Practical limitations to library sizes have been partially addressed recently by improving the proportion of non-redundant or degenerate variants in libraries. For example, the analysis of the frequency and distribution of beneficial single mutants obtained from initial libraries, can be used to define the ratio of templates to be used in recombination by overlap extension PCR [13]. The effect is to improve the diversity of the multiple mutation variants obtained in the shuffled library. Another recent study has examined why libraries constructed using high-mutation frequencies, tend to yield a higher than expected number of functional variants [14]. Increased mutation rates permit the synergistic effects of multiple mutations to be identified more frequently, though they also lead to an increased likelihood of negating positive mutations, or non-functional variants. It was demonstrated that increased mutation rates lead to more unique variants in each library, whereas single mutant libraries can contain many copies of the same variant. This work leads to the possibility of finding an optimal mutagenic load for a given mutagenic method or application.

Another strategy, previously suggested as having the potential to obtain more useful variants from restricted library sizes, is to focus mutations in regions of the enzyme more likely to result in beneficial mutations [4]. In the same review it was also noted that recent examples of the directed evolution of properties traditionally associated with the active site, were producing the majority of mutations in regions that contribute to substrate binding, catalysis or the conformation and dynamics of the active site environment. A more recent and extensive study of previously published rational design and directed evolution experiments demonstrates that indeed by far the majority of mutations that improve the enantioselectivity of enzymes, occur within 10 Å of the enzyme active site [15]. The authors also compared random mutagenesis of five residues in the *P. fluorescens *esterase active site, to single random mutations across the entire enzyme, demonstrating a five-fold improvement of enantioselectivity enhancement for the focussed approach compared to the more random method. In a similar study, four residues which in tetrameric form comprise the sixteen-residue active-site of dihydrofolate reductase (DHFR), were mutated by cassette mutagenesis [16]. The resulting library yielded three mutants with entirely altered active-sites and showing increased activity. More recently, a technique dubbed CASTing (combinatorial active-site saturation), in which pair-wise saturation mutagenesis of residues adjacent in sequence, was focussed into the active site of a lipase from *Pseudomonas aeruginosa*, yielded a number of mutants active on substrates not previously accepted by the wild type [17]. In another striking example, Parikh *et al. *compared the site-saturation mutagenesis of three carefully chosen active-site residues in *E. coli *β-galactosidase, to a previous DNA shuffling experiment for the same enzyme [18]. The previous DNA shuffling experiment enhanced the *k*_cat_/*K*_m _for β-fucosidase by 10-fold after seven rounds, whereas the saturation mutagenesis technique resulted in a 180-fold improvement in a single round. Not all enzyme properties can be expected to improve through active-site mutations alone, however. Indeed, thermostability was shown to be improved equally by mutations close to and distant from the active site [19].

One further promising approach for obtaining more efficient searches of sequence-space is the use of consensus-sequence data for constructing libraries [20]. By aligning the target gene of β-lactamase from *Enterobacter cloacae *with the consensus sequence from 38 homologues, 29 residues were identified as differing from the consensus. Each of these sites was simultaneously mutated back to the consensus sequence, using the QuikChange multi site-directed mutagenesis kit (QCMS) (Stratagene), to produce a combinatorial library. Screening of just 90 variants yielded 15 variants with improved thermostability and subsequent recombination led to further improvements. This demonstrated the potential power of refined library design, though it is yet to be seen whether this type of approach can be applied to properties other than thermostability.

## New screening and selection strategies for directed enzyme evolution

The available methods for screening of, and selection from enzyme libraries have recently been reviewed [21]. New screens are required to enable the identification of improved enzymes from larger libraries, and also to obtain the desired properties with generic methods that measure it directly. The latter issue addresses the often quoted first law of directed evolution, ie. 'you get what you screen for' [22]. A frequent target for the directed evolution of enzymes is the improvement of thermostability which leads to more robust biocatalysts [4], and increased stability in organic solvents, as shown in a recent study on fructose bisphosphate aldolase [23]. Most screens for thermostability have made use of indirect measures, such as resistance to thermoinactivation at high temperatures [23]. Such a screen, though effective in many cases, is not a direct measure of protein stability and is unsuitable for proteins that are reversibly unfolded, or those that are likely to become reversibly unfolded upon mutation [24], thus leading to false positives. To enable a more direct screen for protein stability, the measurement of protein denaturation curves using tryptophan fluorescence in microplates has been explored [25]. The results have shown that using autotitration of denaturant directly into the microwells can yield transition midpoints (C_1/2_) with an accuracy of ± 0.15 M and a throughput of up to 1000 samples per day. Linkage with automated protein purification has the potential to enable application of the screen to directed evolution libraries.

Screening for improved enzyme activity often leads to loss of substrate selectivity (or vice versa). The ability to screen directly for both activity and selectivity would enable more useful enzyme variants to be found. Recently, a cell-surface display approach that uses multiparameter flow cytometry (FACS), demonstrates the benefits of *simultaneously *screening for activity and selectivity, using the *E. coli *endopeptidase Omp T as an example [26]. FACS-based methods permit the screening of up to 10^7 ^cells per hour, enabling large areas of sequence-space to be searched. Combined with the ability to cell sort based upon two different fluorescent reporters, the authors have shown that large numbers of enzyme variants can be screened under simultaneous positive and negative selection pressures to obtain protease mutants with both improved activity to the new Ala-Arg substrate and reduced activity to the Arg-Arg substrate preferred by wild type. Although the particular FACS technique used is limited to cell-surface displayed proteases, the concept of dual screening in this manner could potentially be applied to other enzymes screened by more traditional methods.

The use of selection-based methods has the potential to identify novel enzyme variants from much larger libraries (10^6^-10^13 ^variants), than for screening methods (up to 10^5^-10^7^) and have been reviewed in detail [21,27,28]. Consequently, deeper searches of sequence-space can be performed to access better enzyme variants. Selection of enzymes has been achieved using complementation of the deleted activities in auxotrophic strains [29], enrichment of active beta-lactamase from a background of an inactive point mutant by ribosome display and selection for binding of a mechanism-based inhibitor [30], enrichment of phage display libraries by affinity capture of the phage-bound turnover product [31], and enrichment of phage display libraries by selection for transition-state analogue or suicide inhibitor binding [32-34]. While genetic selection with auxotrophs is limited to the range of activities found to be essential to survival of the host cells, display methods have to potential to explore more novel activities perhaps not previously found in Nature as they rely mostly on the design of good transition-state analogues (TSA) or suicide inhibitors for the desired activity. However, mechanism-based inhibitors do not necessarily represent a full catalytic turnover, and TSA structures may not accurately reflect the true transition state structure in the desired reaction mechanism. Recently, selection for a complete turnover has been achieved using phage display [35]. The product formed after phosphatase turnover is spontaneously converted into an electrophilic reagent that can capture the nearby phage particle. While this approach has enabled significant enhancement of catalytic activity compared to the TSA binding methods it is still to be seen how generally applicable such methods can become and will presumably require a good deal of inventive chemistry to identify suitable capture reagents that spontaneously form after the turnover of other desired reactions.

## Computational approaches for enzyme evolution and design

The last two years have seen a revolution in the use of computational approaches to search sequence space in a combinatorial manner that is analogous to experimental screening of directed evolution libraries. Previously, computational design was used to eliminate the vast proportion of sequences that were incompatible with the protein fold, before experimentally screening the remaining variants for improved activity [36]. The introduction of new activity into protein scaffolds was also achieved using the careful placement of potentially catalytic residues into models and the computational search of variants with improved binding affinity to high-energy reaction intermediates [37,38]. Since these groundbreaking efforts, the use of computational design has expanded to include the thermostabilization of enzymes [39] and the redesign of an enzyme active-site for improved catalytic activity [40]. As computational processing power continues to increase, and protein modelling algorithms become further refined, computational design should soon be capable of tackling more complex enzyme mechanisms and also of dramatically refining experimental library approaches.

## Directed evolution of metabolic pathways

The use of whole cell biocatalysts potentially enables novel molecular synthesis via entire metabolic pathways, involving multiple enzymes. Consequently, directed evolution could also be applied to natural or engineered metabolic pathways, multiple enzyme systems (mini-pathways), and even whole organisms [41]. The directed evolution of the three-enzyme arsenate resistance pathway in *E. coli*, for increased resistance to arsenate, was the first example of using DNA shuffling within a metabolic pathway [42]. Since then, the approach has been combined with that of metabolic engineering to obtain entirely new pathways and products. For example, carotenoids can be synthesised in *E. coli *by expression of genes from the isoprenoid pathways of *Archaeoglobus fulgidus *and *Agrobacterium aurantiacum*. Directed evolution was successfully applied to optimize the expression level of the geranylgeranyl diphosphate (GGPP) synthase gene, increasing the production of astaxanthin [43]. In parallel work, neurosporene was produced by co-exression of isoprenoid pathway genes from *R.sphaeroides*, in *E.coli*. Directed evolution of the *R.sphaeroides *phytoene desaturase yielded variants capable of producing lycopene [44]. A similar independent study yielded a metabolically engineered *E. coli *that could produce lycopene. DNA shuffling of the phytoene desaturase genes from *E. uredovora *and *E. herbicola *resulted in variants of *E. coli *capable of tetradehydrolycopene production. Further extension of the pathway with shuffled lycopene cyclase (crtY) genes from *E. uredovora *and *E. herbicola *yielded the production of torulene [45]. Previously unknown C-45 and C-50 carotenoids have also been synthesised by directed evolution of the C-30 carotenoid synthase (crtM) gene [46]. More recently, a carotenoid desaturase (CrtOx) homolog from *S. aureus *has been coexpressed in the previously evolved tetradehydrolycopene producing strain, to yield the C-40 carotenoid tetradehydrolycopendial [47].

The directed evolution of enzymes within metabolically engineered pathways can benefit product yields via a number of mechanisms, including the reduction of product inhibition at a key enzyme step. Metabolic engineering has been used to enhance glucosamine production in *E. coli *by 15-fold to 60 mg L^-1 ^[48]. The directed evolution by error-prone PCR of the overexpressed glucosamine synthase gene (GlmS), and screening for reduced product inhibition by glucosamine-6-P, yielded an *E. coli *strain capable of producing glucosamine to 17 g L^-1^.

## Conclusion

Directed evolution using genetic techniques has led the way for engineering altered proteins during the last decade, yet there is still considerable scope for developing experimentally simpler and also more efficient techniques and strategies. Recent advances have addressed the issue of library redundancy in terms of the numbers of unique sequences as a function of mutation frequency, and also in terms of focussing random mutagenesis to regions of enzymes more likely to elicit the desired effect. An alternative to improving such library quality is to screen larger libraries by more efficient means. The power of cell surface-display techniques for the selection of novel enzymes is greatly improving the library size and hence the sequence space that can be searched. The ability of this method to perform multiple simultaneous measurements will also potentially improve the quality and usefulness of the enzyme variants isolated from libraries. The use of phage- and ribosome-display methods also has the potential to search much larger variant libraries. While, mechanisms for the affinity-based capture of active enzyme variants are continually being developed and improved, there is still some way to go in widening the general applicability of these methods to more useful enzyme activities.

Computational approaches are also improving rapidly and will become very useful in either creating novel enzymes as starting points for directed evolution, or for defining smarter libraries that contain fewer redundant enzyme variants. While for simple reactions the capability of computational methods is approaching that of genetic techniques, there is still considerable effort required to extend its use towards obtaining novel enzymes with more complex catalytic mechanisms.

Finally, advances in the metabolic engineering of whole cell biocatalysts is stimulating the use of directed evolution to improve metabolic pathways. Considerable progress has been made, especially in the synthesis of novel carotenoids. This area also opens up new targets for directed evolution, such as the reduction of product inhibition on a single enzyme which in turn improves the yield of product from the whole pathway, as demonstrated for the production of glucosamine.

## List of abbreviations

CAST Combinatorial active-site saturation

DHFR Dihydrofolate reductase

FACS Fluorescence-activated cell sorting

GGPP Geranylgeranyl diphosphate

PCR Polymerase chain reaction

QCMS QuikChange multi site

TSA Transition state analogue

## Authors' contributions

EH drafted the manuscript

PAD helped draft and critically revised the manuscript

Both authors have read and approved the final manuscript

## References

[B1] Radzicka A, Wolfenden R (1995). A proficient enzyme. Science.

[B2] Schoemaker HE, Mink D, Wubbolts MG (2003). Dispelling the myths--biocatalysis in industrial synthesis. Science.

[B3] Straathof AJ, Panke S, Schmid A (2002). The production of fine chemicals by biotransformations. Curr Opin Biotechnol.

[B4] Dalby PA (2003). Optimising enzyme function by directed evolution. Curr Opin Struct Biol.

[B5] Hibbert EG, Baganz F, Hailes HC, Ward JM, Lye GJ, Woodley JM, Dalby PA (2005). Directed evolution of biocatalytic processes. Biomolecular Engineering.

[B6] Chen K, Arnold FH (1993). Tuning the activity of an enzyme for unusual environments: sequential random mutagenesis of subtilisin E for catalysis in dimethylformamide. Proc Natl Acad Sci U S A.

[B7] Stemmer WPC (1994). Rapid evolution of a protein in vitro by DNA shuffling. Nature.

[B8] Zhao H, Giver L, Shao Z, Affholter JA, Arnold FH (1998). Molecular evolution by staggered extension process (StEP) in vitro recombination [see comments]. Nat Biotechnol.

[B9] Coco WM, Levinson WE, Crist MJ, Hektor HJ, Darzins A, Pienkos PT, Squires CH, Monticello DJ (2001). DNA shuffling method for generating highly recombined genes and evolved enzymes. Nature Biotech.

[B10] Ostermeier M, Shim JH, Benkovic SJ (1999). A combinatorial approach to hybrid enzymes independent of DNA homology. Nat Biotechnol.

[B11] Miyazaki K, Takenouchi M (2002). Creating random mutagenesis libraries using megaprimer PCR of whole plasmid. Biotechniques.

[B12] Wang CL, Yang DC, Wabl M (2004). Directed molecular evolution by somatic hypermutation. Protein Engineering Design & Selection.

[B13] Hamamatsu N, Aita T, Nomiya Y, Uchiyama H, Nakajima M, Husimi Y, Shibanaka Y (2005). Biased mutation-assembling: an efficient method for rapid directed evolution through simultaneous mutation accumulation. Protein Engineering Design & Selection.

[B14] Drummond DA, Iverson BL, Georgiou G, Arnold FH (2005). Why high-error-rate random mutagenesis libraries are enriched in functional and improved proteins. J Mol Biol.

[B15] Park S, Morley KL, Horsman GP, Holmquist M, Hult K, Kazlauskas RJ (2005). Focusing mutations into the P. fluorescens esterase binding site increases enantioselectivity more effectively than distant mutations. Chemistry & Biology.

[B16] Schmitzer AR, Lepine F, Pelletier JN (2004). Combinatorial exploration of the catalytic site of a drug- resistant dihydrofolate reductase: creating alternative functional configurations. Protein Engineering Design & Selection.

[B17] Reetz MT, Bocola M, Carballeira JD, Zha DX, Vogel A (2005). Expanding the range of substrate acceptance of enzymes: Combinatorial active-site saturation test. Angewandte Chemie-International Edition.

[B18] Parikh MR, Matsumura I (2005). Site-saturation mutagenesis is more efficient than DNA shuffling for the directed evolution of b-fucosidase from b-galactosidase. J Mol Biol.

[B19] Morley KL, Kazlauskas RJ (2005). Improving enzyme properties: when are closer mutations better?. Trends in Biotechnology.

[B20] Amin N, Liu AD, Ramer S, Aehle W, Meijer D, Metin M, Wong S, Gualfetti P, Schellenberger V (2004). Construction of stabilized proteins by combinatorial consensus mutagenesis. Protein Engineering Design & Selection.

[B21] Aharoni A, Griffiths AD, Tawfik DS (2005). High-throughput screens and selections of enzyme-encoding genes. Current Opinion in Chemical Biology.

[B22] Schmidt-Dannert C, Arnold FH (1999). Directed evolution of industrial enzymes. Trends Biotechnol.

[B23] Hao JJ, Berry A (2004). A thermostable variant of fructose bisphosphate aldolase constructed by directed evolution also shows increased stability in organic solvents. Protein Engineering Design & Selection.

[B24] Gray KA, Richardson TH, Kretz K, Short JM, Bartnek F, Knowles R, Kan L, Swanson PE, Robertson DE (2001). Rapid evolution of reversible denaturation and elevated melting temperature in a microbial haloalkane dehalogenase. Advanced Synthesis & Catalysis.

[B25] Aucamp JP, Cosme AM, Lye GJ, Dalby PA (2005). High-throughput measurement of protein stability in microtiter plates. Biotechnology and Bioengineering.

[B26] Varadarajan N, Gam J, Olsen MJ, Georgiou G, Iverson BL (2005). Engineering of protease variants exhibiting high catalytic activity and exquisite substrate selectivity. Proc Natl Acad Sci U S A.

[B27] Forrer P, Jung S, Pluckthun A (1999). Beyond binding: using phage display to select for structure, folding and enzymatic activity in proteins. Curr Opin Struct Biol.

[B28] Fernandez-Gacio A, Uguen M, Fastrez J (2003). Phage display as a tool for the directed evolution of enzymes. Trends in Biotechnology.

[B29] Evnin LB, Vasquez JR, Craik CS (1990). Substrate specificity of trypsin investigated by using a genetic selection. Proc Natl Acad Sci U S A.

[B30] Amstutz P, Pelletier JN, Guggisberg A, Jermutus L, Cesaro-Tadic S, Zahnd C, Pluckthun A (2002). In vitro selection for catalytic activity with ribosome display. Journal of the American Chemical Society.

[B31] Demartis S, Huber A, Viti F, Lozzi L, Giovannoni L, Neri P, Winter G, Neri D (1999). A strategy for the isolation of catalytic activities from repertoires of enzymes displayed on phage. J Mol Biol.

[B32] Arkin MR, Wells JA (1998). Probing the importance of second sphere residues in an esterolytic antibody by phage display. J Mol Biol.

[B33] Goud GN, Artsaenko O, Bols M, Sierks M (2001). Specific glycosidase activity isolated from a random phage display antibody library. Biotechnol Prog.

[B34] Widersten M, Mannervik B (1995). Glutathione Transferases with Novel Active-Sites Isolated by Phage Display from A Library of Random Mutants. Journal of Molecular Biology.

[B35] Cesaro-Tadic S, Lagos D, Honegger A, Rickard JH, Partridge LJ, Blackburn GM, Pluckthun A (2003). Turnover-based in vitro selection and evolution of biocatalysts from a fully synthetic antibody library. Nature Biotechnology.

[B36] Hayes RJ, Bentzien J, Ary ML, Hwang MY, Jacinto JM, Vielmetter J, Kundu A, Dahiyat BI (2002). Combining computational and experimental screening for rapid optimization of protein properties. Proc Natl Acad Sci U S A.

[B37] Bolon DN, Mayo SL (2001). Enzyme-like proteins by computational design. Proc Natl Acad Sci U S A.

[B38] Dwyer MA, Looger LL, Hellinga HW (2004). Computational design of a biologically active enzyme. Science.

[B39] Korkegian A, Black ME, Baker D, Stoddard BL (2005). Computational thermostabilization of an enzyme. Science.

[B40] Lassila JK, Keeffe JR, Oelschlaeger P, Mayo SL (2005). Computationally designed variants of Escherichia coli chorismate mutase show altered catalytic activity. Protein Engineering Design & Selection.

[B41] Schmidt-Dannert C (2001). Directed evolution of single proteins, metabolic pathways, and viruses. Biochemistry.

[B42] Crameri A, Dawes G, Rodriguez EJ, Silver S, Stemmer WP (1997). Molecular evolution of an arsenate detoxification pathway by DNA shuffling [see comments]. Nat Biotechnol.

[B43] Wang CW, Oh MK, Liao JC (2000). Directed evolution of metabolically engineered Escherichia coli for carotenoid production. Biotechnology Progress.

[B44] Wang CW, Liao JC (2001). Alteration of product specificity of Rhodobacter sphaeroides phytoene desaturase by directed evolution. Journal Of Biological Chemistry.

[B45] Schmidt-Dannert C, Umeno D, Arnold FH (2000). Molecular breeding of carotenoid biosynthetic pathways. Nat Biotechnol.

[B46] Umeno D, Arnold FH (2004). Evolution of a pathway to novel long-chain carotenoids. Journal of Bacteriology.

[B47] Mijts BN, Lee PC, Schmidt-Dannert C (2005). Identification of a carotenoid oxygenase synthesizing acyclic xanthophylls: Combinatorial biosynthesis and directed evolution. Chemistry & Biology.

[B48] Deng MD, Severson DK, Grund AD, Wassink SL, Burlingame RP, Berry A, Running JA, Kunesh CA, Song L, Jerrell TA, Rosson RA (2005). Metabolic engineering of Escherichia coli for industrial production of glucosamine and N-acetylglucosamine. Metabolic Engineering.

